# Diagnostic Utility of Immunohistochemical Detection of MEOX2, SOX11, INSM1 and EGFR in Gliomas

**DOI:** 10.3390/diagnostics13152546

**Published:** 2023-07-31

**Authors:** Jiri Soukup, Lucie Gerykova, Anjali Rachelkar, Helena Hornychova, Michael Christian Bartos, Petr Krupa, Barbora Vitovcova, Zuzana Pleskacova, Petra Kasparova, Katerina Dvorakova, Veronika Skarkova, Jiri Petera

**Affiliations:** 1 Department of Pathology, Military University Hospital Prague, U Vojenske Nemocnice 1200, Praha 6, 169 02 Prague, Czech Republic; 2 The Fingerland Department of Pathology, Charles University, Faculty of Medicine in Hradec Králové and University Hospital Hradec Králové, Sokolska 581, 500 05 Hradec Kralove, Czech Republic; 3 Department of Oncology and Radiotherapy, Charles University, Faculty of Medicine in Hradec Králové and University Hospital Hradec Králové, Sokolska 581, 500 05 Hradec Kralove, Czech Republic; 4 Department of Neurosurgery, Charles University, Faculty of Medicine in Hradec Králové and University Hospital Hradec Králové, Sokolska 581, 500 05 Hradec Kralove, Czech Republic; 5 Department of Neuroregeneration, Institute of Experimental Medicine, Czech Academy of Sciences, 142 20 Prague, Czech Republic; 6 Department of Medical Biology and Genetics, Charles University, Faculty of Medicine in Hradec Králové, Zborovská 2089, 500 03 Hradec Kralove, Czech Republic; vitovcob@lfhk.cuni.cz (B.V.);

**Keywords:** MEOX2, SOX11, glioblastoma, EGFR, INSM1, CD34

## Abstract

Histological identification of dispersed glioma cells in small biopsies can be challenging, especially in tumours lacking the *IDH1* R132H mutation or alterations in TP53. We postulated that immunohistochemical detection of proteins expressed preferentially in gliomas (EGFR, MEOX2, CD34) or during embryonal development (SOX11, INSM1) can be used to distinguish reactive gliosis from glioma. Tissue microarrays of 46 reactive glioses, 81 glioblastomas, 34 IDH1-mutant diffuse gliomas, and 23 gliomas of other types were analysed. Glial neoplasms were significantly more often (*p* < 0.001, χ^2^) positive for EGFR (34.1% vs. 0%), MEOX2 (49.3% vs. 2.3%), SOX11 (70.5% vs. 20.4%), and INSM1 (65.4% vs. 2.3%). In 94.3% (66/70) of the glioblastomas, the expression of at least two markers was observed, while no reactive gliosis showed coexpression of any of the proteins. Compared to IDH1-mutant tumours, glioblastomas showed significantly higher expression of EGFR, MEOX2, and CD34 and significantly lower positivity for SOX11. Non-diffuse gliomas were only rarely positive for any of the five markers tested. Our results indicate that immunohistochemical detection of EGFR, MEOX2, SOX11, and INSM1 can be useful for detection of glioblastoma cells in limited histological samples, especially when used in combination.

## 1. Introduction

Evaluation of small brain biopsies for the presence of glioma is among the most challenging tasks in routine surgical neuropathology. Often, nonspecific reactive gliosis may simulate neoplasia, or the infiltration is subtle and cannot be reliably confirmed on morphologic grounds. Therefore, in some cases, the distinction is extremely difficult or even impossible, while it bears grave clinical significance.

In recent years, the diagnosis of a subset of gliomas carrying the *IDH1* R132H mutation (IDH1mt) has been made easy and reproducible due to the routine use of a mutation specific antibody [1]. However, the use of the anti-IDH1 R132H antibody has its limitations; although most oligodendrogliomas and low-grade astrocytomas carry the *IDH1* R132H mutation, other mutations occur in *IDH1* or *IDH2* genes [2,3] that cannot be detected with the antibody. In the case of glioblastomas (GBMs), the situation is more complicated. Although around 12% carry *IDH1/2* alterations like their low-grade counterparts [4], the majority of GBMs show a different genetical background [5]. In the recent WHO classification [6], this subset of GBMs was eventually merged with the category of *IDH*-mutated astrocytomas, given the shared biological and clinical characteristics. The only other antibody used routinely for the detection of gliomas in biopsies is anti-p53, with glioma cells generally showing strong immunoreactivity [7,8]. The immunoreactivity of p53 corresponds to the presence of *TP53* mutations and, similar to *IDH1* R132H, only a subset of gliomas carry this alteration. Although there are other immunohistochemically exploitable genetic alterations in gliomas (i.e., *BRAF* V600E mutation, histone 3 K27M mutation), their frequency is very low and limited to specific entities [7]. Thus, it would be of great clinicopathologic importance to identify additional proteins that could help to distinguish between reactive gliosis and minimal infiltration by glioma cells in limited samples.

In the past, several proteins were identified with differential expression in embryonal brain and/or tumour tissue when compared to the normal adult brain. This includes epidermal growth factor receptor (EGFR), a transmembrane receptor protein localised on chromosome 7 that is expressed in neural stem cells of subventricular zone [9] and in a subset of glioblastomas with *EGFR* gene amplification or mutation [5]. EGFR signalling promotes glioma cell survival, proliferation, and migration by an activation of PI3K/Akt or MAPK signalling pathways [10]. Another protein, mesenchyme homeobox 2 (MEOX2), is often overexpressed in glioblastomas. Similar to the *EGFR* gene, *MEOX2* is localised on chromosome 7, and gains of chromosome 7 can, in part, explain its expression in a subset of tumours [11]. The literature on MEOX2 in glioblastomas is scarce; however, there are data that indicate that MEOX2 functions as a transcription factor involved in the regulation of viability, proliferation, and differentiation of glioma cells by interacting with PI3K/Akt and MAPK pathways [12]. INSM1 and SOX11 are other transcription factors. Both are necessary for normal neurogenesis in the embryonal and adult brain [13,14,15]. INSM1 is not detected in the adult brain tissue samples but it is present in some gliomas [16]. Under normal conditions, INSM1 expression is seen in a subset of neuronal precursors in basal zones of neuroepithelium, where it is responsible for positive regulation of cell proliferation [17]. In gliomas, however, its role is not well understood. SOX11 is a transcription factor necessary for sustained proliferation of neural progenitor cells (NPCs), acting by upregulation of Nmyc transcription factor [14]. SOX11 was detected in various proportions of gliomas [18,19] and there are data suggesting a positive correlation between SOX11 expression and patient survival [18], possibly as a result of plagl1 protein inhibition and induction of cell differentiation [20]. Lastly, CD34 is a transmembrane glycoprotein involved in cellular adhesion [21]. In the normal brain, CD34 is not detectable; however, it is commonly positive in low grade glioneuronal tumours and malformations associated with epilepsy and in a subset of glioblastomas [22,23].

In this study, we decided to evaluate the immunohistochemical expression of selected proteins in a cohort of reactive glioses and various glial tumours of different grades. We were interested in proteins that are known to be expressed in subsets of malignant glial tumours: while some proteins have been studied for this purpose in the past (EGFR) [24], others have been mostly studied in unrelated pathological processes (INSM1, SOX11, CD34) or have been studied only rarely in the context of diagnostic pathology (MEOX2). 

## 2. Materials and Methods

### 2.1. Patients and Selection of Cases

The design of the study was approved by the local ethics committee of the first author’s institution. The archive of The Fingerland Department of Pathology was searched for cases diagnosed between 2012 and 2020. For the reactive gliosis group (RG group), all the cases diagnosed as “reactive gliosis” or having the “gliosis” term in the text of the biopsy report were reviewed. Only cases with a representative amount of reactive glial tissue (n = 46) were selected for the study; cases with gliosis and concurrent glial neoplasms were excluded. The cause of gliosis included metastasis (n = 13), vascular malformation (n = 13), abscess (n = 7), lymphoma (n = 6), meningioma (n = 3), and one case each of hematoma, meningeal Rosai–Dorfman disease, craniopharyngioma, and old infarction. For the group of *IDH1* mutant astrocytomas (n = 17; grade 2 n = 10; grade 3 n = 3; grade 4 n = 4), only immunoreactive cases with *IDH1* R132H antibody were selected. For the group of oligodendroglial tumours (n = 17; grade 2 n = 12; grade 3 n = 5), only 1p/19q codeleted cases were selected. Additional representative examples of ependymomas (n = 10, including 2 subependymomas), pilocytic astrocytomas (n = 8 including one pilomyxoid astrocytoma), and gangliogliomas (n = 5) were included. For the glioblastoma group (GBM group, n = 81), all glioblastoma cases diagnosed between 2012 and 2015 at our institution were reviewed and only cases with a representative amount of tumour tissue and without previous therapy were selected. In all selected tumour cases, the original diagnosis was reviewed and confirmed by a single experienced neuropathologist (J.S.) according to the current (2021) WHO Classification. The samples of both neoplastic and non-neoplastic processes originated from different brain areas, according to available material from the surgery. The study was performed in accordance with the Declaration of Helsinki and it was approved by ethical committee of the first author’s institution (Ethical committee decision reference no. 202104 P04, 8 April 2021).

### 2.2. Tissue Handling and Construction of Tissue Microarrays

**Figure 1:**
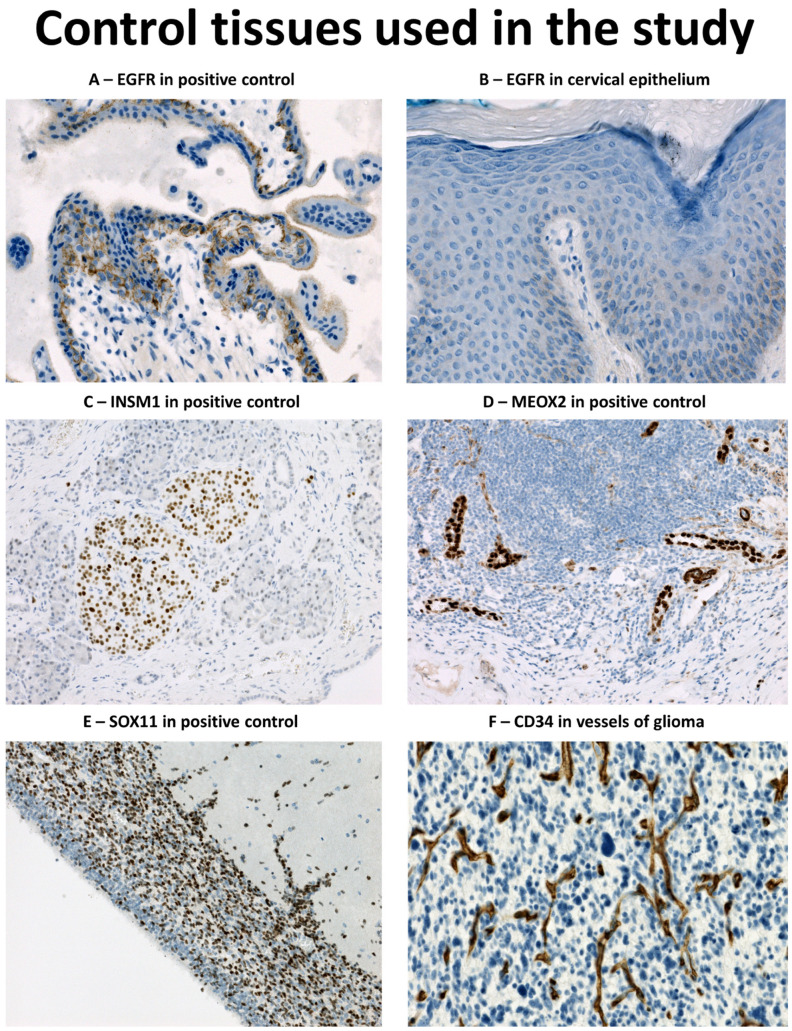
Positive controls used for immunohistochemical analysis: (**A**) Placenta cytotrophoblast and syncytiotrophoblast with distinct membranous staining for EGFR (EGFR immunohistochemistry, original magnification 200×); (**B**) Faint or absent immunoreactivity of EGFR in normal skin (EGFR immunohistochemistry, original magnification 200×); (**C**) Pancreatic isles positive for INSM1 (INSM1 immunohistochemistry, original magnification 200×); (**D**) High endothelial venules in MALT tissue of an appendix positive for MEOX2 (MEOX2 immunohistochemistry, original magnification 200×); (**E**) Neuronal precursors in the germinal matrix of fetal brain positive for SOX11 (SOX11 immunohistochemistry, original magnification 200×); (**F**) Blood vessels in glioma positive for CD34 (CD34 immunohistochemistry, 200×).

Archival tissue in the form of formalin-fixed paraffin embedded (FFPE) blocks was used for the analysis. The tissue microarrays (TMAs) were constructed using TMA Master II system (3DHISTECH Ltd., Budapest, Hungary). For each case, three tissue cores (1 mm in diameter) representative of tumour/gliosis were transferred from donor blocks into the recipient block. In total, 8 TMAs were constructed. For the validation step, selected gliosis blocks were cut and stained “whole-section” (WS); for both WSs and TMAs, 2-μm-thick sections were cut and routine H and E staining and immunohistochemical studies were performed. Normal samples of skin, tonsils, and cervix were used for the immunohistochemical assay to assess its sensitivity (Figure 1A,B).

### 2.3. Immunohistochemical Analysis and In Situ Hybridisation (ISH) for EGFR

The list of antibodies and relevant details of immunohistochemical protocols are summarized in Table 1. Heat induced epitope retrieval (HIER) was used for antigen retrieval, employing different pH according to the antibody. For EGFR, a proteolytic pretreatment step was used. Section staining was carried out on Benchmark Ultra stainer (Ventana/Roche, Tucson, AZ, USA) using either the Ventana ultraView Universal DAB detection kit or the Ventana OptiView DAB IHC detection kit; both methods use the avidin-biotin complex method with horseradish peroxidase as an enzyme and DAB (3,3′-diaminobenzidine) as a chromogen. An Agilent/Dako Autostainer 48 (Agilent, Santa Clara, CA, USA) using the PT-Link pretreatment system and the EnVision Flex detection kit with DAB was used for INSM1. Subsequently, all slides were counterstained with haematoxylin. Normal placental tissue was used to set up EGFR assay. Positive control on slide was used with different tissues according to the antigen detected antigen (Figure 1). For *EGFR* amplification detection in 4 TMAs with GBM, silver ISH with EGFR DNA Probe (Ventana/Roche) and ultraView SISH Detection Kit (Ventana/Roche) was employed on a Benchmark Ultra stainer. Additional GBMs cases without enough representative material in TMA were evaluated as whole sections using the ZytoLight SPEC EGFR/CEN 7 Dual Color Probe set (ZytoVision, Bremehaven, Germany).

### 2.4. Morphological Evaluation

All TMAs were evaluated independently by two experienced neuropathologists (J.S. and P.K.) for the presence of reactive gliosis or glioma tissue, using H and E and GFAP stained slides. Only TMA cases with at least one representative core were included for further analyses. Immunohistochemistry results were first digitalised using a Leica Aperio AT2 slide scanner (Leica Biosystems, Buffalo Grove, IL, USA) and then evaluated with Aperio ImageScope software (version 12.1, Leica Biosystems, Buffalo Grove, IL, USA). Immunohistochemistry evaluation was performed independently by two observers (J.S. and L.G. or A.R.). The percentage of positive cells and the most prevalent staining intensity (1—weak, 2—moderate, 3—strong) was observed. Cases with positive cells were considered positive for the purposes of the study. The cases with discordant (positive vs. negative) results were discussed at the multi-headed microscope until consensus was reached. The average percentage of positive cells and the modified H score (mHS) were used for the analysis. The modified H score (mHS) was counted as the average intensity * the average percentage of positive cells, reaching values between 0 and 300. *EGFR* amplification was assessed by a single pathologist (J.S.) using Nikon Eclipse Ci optical microscope (Nikon, Minato, JP) with fluorescence extension and reported either as present or absent. The results of immunohistochemistry and genetics in individual cases are available in Supplementary Table S1.

### 2.5. Statistical Analysis

Statistical analysis was performed using SigmaPlot 14 software (version 11, Systat Software, San Jose, CA, USA). The Shapiro–Wilk normality test was used to evaluate the distribution of the data. Non-parametric data were reported using median and interquartile range (IQR). Two-tailed versions of Student t-test, ANOVA, Mann–Whitney-u (MW) test, Kruskal–Wallis (KW) test, and χ^2^ test were used to analyse the data; *p*-values < 0.01 (Bonferroni correction for testing five different immunohistochemical markers) were considered statistically significant.

## 3. Results

### 3.1. Immunohistochemical Profile of Reactive Gliosis

**Figure 2:**
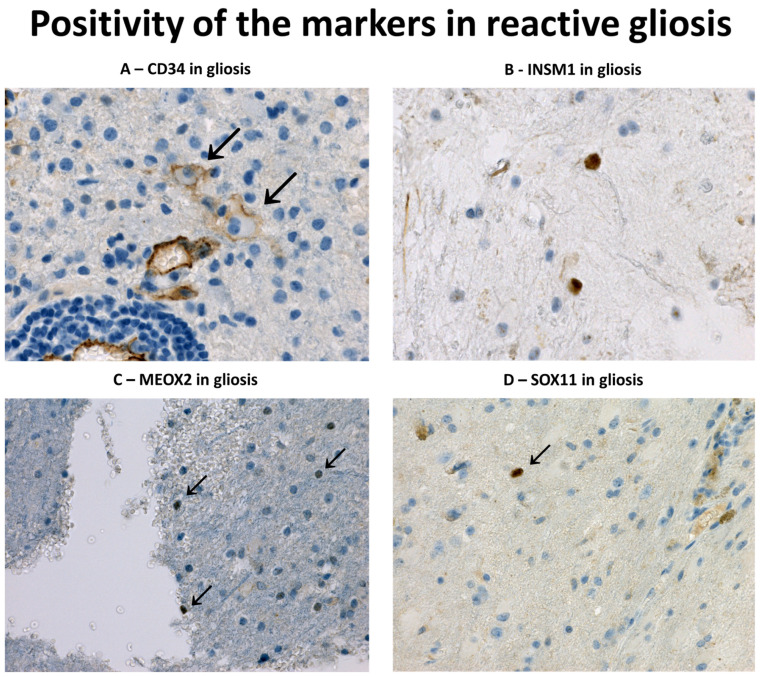
Positivity of the markers studied in reactive glial tissue: (**A**) CD34 immunoreactivity of scattered gemistocytes (arrows) in a sample of CNS DLBCL pretreated with glucocorticoids (CD34 immunohistochemistry, original magnification 400×); (**B**) Rare INSM1+ cells were observed adjacent to metastasis of carcinoma in one case (INSM1 immunohistochemistry, original magnification 400×); (**C**) Rare cells weakly positive for Meox2 in a tissue surrounding an AVM (MEOX2 immunohistochemistry, original magnification 400×); (**D**) Dispersed SOX11+ cells were observed in several reactive condition, like in a case of carcinoma metastasis (SOX11 immunohistochemistry, original magnification 400×).

Positivity rates of individual markers in TMAs with gliosis and glioma cases are noted in Table 2. We did not observe immunoreactivity of EGFR in gliosis and only a single case was positive for INSM1 (metastasis, 1% of cells, mHS = 1) and MEOX2 (vascular malformation, 1% of cells, mHS = 2). SOX11 was weakly positive (range 1–1.5%, mHS 1–1.5%) in nine cases and CD34 was positive in five cases (range 3–5%, mHS 3–10). The immunoreactivity of individual markers in glioses is depicted in Figure 2. We did not observe coexpression of two or more markers in any of the gliosis case. Compared to glioses, glial neoplasms were significantly more often positive (*p* < 0.001, χ^2^) for EGFR (33.8%), MEOX2 (48.9%), SOX11 (69.9%) and INSM1 (64.9%). The sensitivity and specificity of the negative staining for the diagnosis of gliosis were as follows: EGFR—100% sensitive, 32.8% specific; MEOX2—97.7% sensitive, 37.5% specific; SOX11—79.5% sensitive, 46.6% specific and INSM1—97.7% sensitive, 47.8% specific. After excluding IDH1 R132H mutant gliomas from the analysis, the specificity increased to 41.5% for EGFR, 53.2% for MEOX2, 50.7% for SOX11 and 57.3% for INSM1.

To further validate the results, we performed WS staining (MEOX2, EGFR, INSM1, and SOX11) on nine cases included in TMA. We observed single dispersed (<1%) SOX11-positive cells in two cases (gliosis around invasive meningioma and abscess), showing weak to strong immunoreactivity. The remaining WSs were devoid of positive cells.

### 3.2. Immunohistochemical Profile of Diffuse Gliomas with IDH1 Mutation

**Figure 3:**
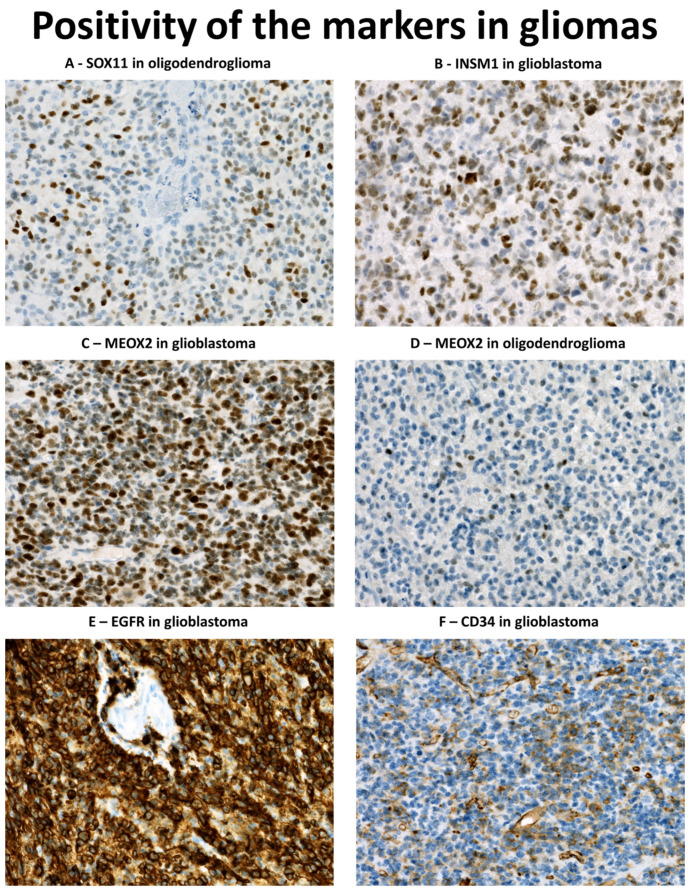
Positivity of the markers studied in diffuse gliomas: (**A**) SOX11 immunoreactivity in a case of a grade 3 oligodendroglioma (SOX11 immunohistochemistry, original magnification 200×); (**B**) INSM1 immunoreactivity in a glioblastoma (INSM1 immunohistochemistry, original magnification 200×); (**C**) Strong and diffuse MEOX2 immunoreactivity in *EGFR*-amplified glioblastoma (MEOX2 immunohistochemistry, original magnification 200×); (**D**) Weak and focal MEOX2 immunoreactivity in a grade 3 oligodendroglioma (MEOX2 immunohistochemistry, original magnification 200×); (**E**) Strong EGFR immunoreactivity in *EGFR*-amplified glioblastoma (EGFR immunohistochemistry, original magnification 200×); (**F**) CD34 immunoreactive cells in a glioblastoma (CD34 immunohistochemistry, original magnification 200×).

The mHS in different glial tumour subgroups are shown in Table 3. We observed a consistent positivity for SOX11 in the *IDH1mt* subset of diffuse gliomas (n = 34, Figure 3A), and IDH1mt tumours showed significantly higher expression of SOX11 when compared to glioblastomas (median mHS 2 vs. 25.8, *p* < 0.001, MW). However, there was no difference in SOX11 between astrocytic (1p/19q intact) and oligodendrocytic (1p/19q co-deleted) tumours. Higher SOX11 expression in *IDH1mt* tumours was significantly associated with tumour grade (median mHS G2 = 7.5, G3 = 55, G4 = 47.6, *p* < 0.001, KW). INSM1 expression did not differ between astrocytic and oligodendroglial tumours but it was significantly higher in grade 4 astrocytomas when compared to grade 2 and 3 tumours (median mHS 86.3 vs. 4 and 1.5, *p* = 0.007, KW, Figure 3B). Compared to the GBM group, INSM1 immunoreactivity did not differ significantly (*p* = 0.14, MW). The expression of MEOX2 (Figure 3C), EGFR, and CD34 was significantly higher in GBMs (*p* < 0.002, MW). MEOX2 was detected only in one astrocytoma (grade 2, 1% of cells, mHS 2) and one oligodendroglioma (grade 3, 8.5% of cells, mHS 12.75, Figure 3D). EGFR was detected in 20.6% (7/34) of *IDH1mt* tumours including four astrocytomas (all grade 2) and three oligodendrogliomas (grade 2 n = 2, grade 3 n = 1). In all the cases, EGFR staining intensity was weak and limited to 1–5.5% of cells.

### 3.3. Immunohistochemical Profile of Glioblastomas

Data on *EGFR* amplification were available in 79 GBM. Of these, 40.5% (32/79) harboured *EGFR* amplification. Amplified cases showed significantly higher expression of EGFR (medians mHS 0 vs. 160, *p* < 0.001, MW, Figure 3E) and MEOX2 (medians mHS 3 vs. 40, *p* < 0.001, MW, Figure 3C); we did not observe an association between *EGFR* status and SOX11, INSM1 or CD34 (Figure 3F). In 70 cases, the immunohistochemical expression of the five markers could be assessed. Expression of a single marker was observed in 5.7% (4/70), two markers were positive in 25.7% (18/70, most commonly SOX11/INSM1, n = 8 and EGFR/MEOX2, n = 5), three markers were positive in 22.9% (16/70, most commonly MEOX2/SOX11/INSM1, n = 9), and four markers were positive in 42.9% (30/70, most commonly MEOX2/SOX11/EGFR/INSM1, n = 20). In 2.9% (2/70), all five markers were positive. All the tumours (n = 70) were stained with at least one marker.

### 3.4. Immunohistochemical Profile of Non-Diffuse Glial Tumours in the Study

Immunoreactivity of studied markers was observed only rarely in group of non-diffuse gliomas. EGFR was consistently negative, MEOX2 was positive in a single ganglioglioma (1% of cells, mHS = 2), SOX11 in one PA (1% of cells, mHS = 1) and ependymoma (10% of cells, mHS = 10), CD34 in one pilocytic astrocytoma (1% of cells, mHS = 2), ependymoma (1% of cells, mHS = 1), subependymoma (20% of cells, mHS = 40) and three gangliogliomas (range 1–16% of cells, mHS 1–24). INSM1 was positive in one pilocytic astrocytoma (2% of cells, mHS = 2) and 4 gangliogliomas (range 1–3.15% of cells, range mHS 1–9.5). Coexpression of INSM1 and CD34 was seen in 1 PA and 3 GGs; one of these GGs also coexpressed MEOX2. In remaining tumours, no coexpression of multiple markers was observed.

## 4. Discussion

Glial neoplasms have been shown to express a diverse array of proteins that are not normally detected in adult brain tissue. These include EGFR [24,25,26,27], commonly associated with amplification of the gene [25,26], SOX11 [18,19,28] and INSM1 [16], involved in different stages of normal embryonal neurogenesis [28,29], MEOX2 [11], associated with a poor prognosis and a subset of mesenchymal subtype GBMs [30], and CD34, an adhesive molecule detected in a subset of low grade epilepsy associated glioneuronal tumours [22], pleomorphic xanthoastrocytomas [31], and a subset of GBMs [32]. However, only some of these proteins have been studied in reactive glial proliferations [16,24,33]. The use of the IDH1 R132H antibody significantly facilitated the diagnosis of *IDH1mt* tumours in histological sections, even when only scattered tumour cells are present; thus, these cases do not present a diagnostic dilemma in most cases. However, most glial neoplasms, including GBM, lack the *IDH1* mutation. *IDH1mt* GBMs, strongly associated with neuronal/proneuronal subsets of GBMs [5], are nowadays classified as a part of spectrum of *IDH1mt* astrocytomas (Astrocytoma, *IDH1*-mutated, CNS WHO grade 4); we therefore analysed them in the group with other *IDH1mt* astrocytomas [6].

*EGFR* amplification or activating mutation is an oncogenic driver in GBMs, designated as a so-called classical subtype [5]. The subtype comprises between 26 and 42% of GBMs in all studies, using different datasets [5,25,27]. This is similar to 40.2% of GBMs with *EGFR* amplification in our cohort. EGFR immunoreactivity was significantly associated with gene amplification status [25,26], which is consistent with our findings. EGFR immunoreactivity was also observed in 20.6% (7/34) of *IDH1mt* tumours, although weak and limited, and unrelated to tumour grade. EGFR immunoreactivity in *IDH1mt* tumours was also observed in past [27,33], although these usually do not usually harbour *EGFR* amplification [34,35]. In our study, the lack of EGFR immunoreactivity was the only 100% sensitive marker of reactive gliosis, although with the lowest specificity, since only 31.9% (43/135) of all gliomas showed any immunoreactivity. In a previous study, EGFR positivity was found in 88% (22/25) of glioses, although it was weak and often limited in contrast to strong positivity in diffuse gliomas [33]. We are convinced that differences in EGFR immunohistochemical detection are responsible for this discrepancy. DAKO EGFR pharmDx kit was used in previous study [33] and, according to the kit documentation, membranous EGFR immunoreactivity can be observed among others in normal squamous epithelia of cervix, tonsil, and skin. In our study, we observed consistent membranous positivity of cytotrophoblast cells and the surface membrane of syncytiotrophoblast in a placenta included as the positive control, while detecting no or very faint immunoreactivity in normal samples of the cervix, tonsil, and skin. According to Human Protein Atlas (proteinatlas.org, accessed on 12 December 2022) [36], placenta shows the highest levels of expression of *EGFR* mRNA when compared to any other normal human tissue, with the skin showing second highest. Therefore, the EGFR assay in this study yielded much lower sensitivity in order to detect only high EGFR expressors, typically *EGFR* amplified tumour cells. This was also reflected by the strong positivity of EGFR in glioma cells when compared to weak immunoreactivity of glioses documented previously [33]. In our opinion, EGFR immunohistochemistry can be reliably used for the detection of dispersed glioma cells; however, the assay must be calibrated for the purpose (positive immunoreactivity in membrane of trophoblastic cells and negative reaction with epidermis cells) and validated using at least several samples of glioses and gliomas. This underlies the importance of a fit-for-purpose positive control used to establish the positivity threshold in an immunohistochemical assay, similar to TFE3 [37].

Another marker studied, MEOX2, has been also associated with classical subtype GBMs [11,38], although to a lesser extent, as its expression was also documented in mesenchymal GBMs [30]. The *MEOX2* gene is located at the 7p21 locus and its overexpression in gliomas is associated with chromosome 7 gains [11], a hallmark alteration in classical subtype GBMs [5]. In GBM, MEOX2 overexpression did not show prognostic significance, although it was associated with a worse outcome in diffuse gliomas in general [11]. In our cohort, MEOX2 immunoreactivity was significantly higher in the subset of *EGFR*-amplified GBMs (96.7%, 29/30); it was commonly seen in the rest of the GBMs (70.2%, 33/47) and only rarely observed in IDH1mt tumours (5.9%, 2/34) and non-diffuse gliomas (4.3%, 1/23). These findings are consistent with a previous report of low expression in IDH1mt tumours [11]. In glioses, single MEOX2-positive cells were observed in only one TMA case and none of the cases analysed as whole section, demonstrating exceedingly rare immunoreactivity in various reactive glial processes.

SOX11 plays an important role in normal neurogenesis [14,28], participating in the early differentiation of neural stem cells into immature neurons [15]. In addition, SOX11 regulates the early development of oligodendrocytes, being expressed in oligodendrocyte progenitor cells (OPC) [39]. In adult brain tissue, SOX11 is not expressed, with the exception of areas of neurogenesis [15,19,28,40]. SOX11 was previously reported in a varying number of glial neoplasms, ranging from 93–100% of astrocytomas, using polyclonal antibody [18], to a more limited 50% of gliomas when monoclonal antibody MRQ-58 was used. One study demonstrated a higher expression of SOX11 in *IDH1mt* tumours [40]; this was not observed in another [18]. In our study, SOX11 was the only marker significantly associated with the IDH1mt subgroup, regardless of 1p/19q status. Interestingly, IDH-mutant tumours were strongly associated with proneural/neural/OPC gene expression signatures [5,41,42]; this may explain the more common immunoreactivity of SOX11 in the subset. SOX11 was also the most commonly positive marker in reactive glial tissue in TMAs (20.9%) and the only marker observed in WSs, although the expression was limited to single positive cells. It is questionable whether these occasional positive cells in areas of injury represent differentiating OPCs, which are known to participate in reparative processes after brain injury [43]. Similar to SOX11, INSM1 is another transcriptional factor important for early neurogenesis [13] and that is lacking in the normal adult brain [16]. Consistently, only one INSM1-positive case was observed among glioses in TMA when compared to immunoreactivity in 65% of gliomas. GBMs, oligodendrogliomas, and gangliogliomas were positive in almost 80% of cases, followed by 35.5% of astrocytomas, while ependymomas and pilocytic astrocytomas were almost always negative, as in the previous report [16]. CD34 was only rarely observed in GBMs and other gliomas in our study, with the exception of GGs; this limits its practical use. Interestingly, we observed CD34 in five glioses (two AV malformations, a lymphoma, a chronic hematoma, and a metastasis of carcinoma). This was an unexpected finding, given CD34 paucity in the normal brain [22]. CD34-positive hematopoietic stem cells were reported in glial tumours [44] and also in subsets of proliferating microglias of rat models of amyotrophic lateral sclerosis [45]. We are unsure whether the positive cells in gliotic tissue really belonged to the glial lineage; a double immunohistochemistry assay would be necessary to confirm that. However, for practical purposes, CD34 should be interpreted with care, and only when unequivocal glial morphology can be recognised.

Overexpression of EGFR was not observed in reactive tissue, while MEOX2 and INSM1 were observed in single cases only. Of the five markers studied, only SOX11 was observed in occasional cells more frequently, while the lack of CD34 in the majority glial tumours limited its potential practical use. Importantly, we did not observe coexpression of any of the studied proteins in reactive glioses. However, this was commonly observed in GBMs, the most significant category of tumours in differential diagnosis. Therefore, the identification of single positive cells in a small brain biopsy sample with expression of EGFR, MEOX2, or INSM1, or coexpression of two or more of these (including SOX11 and CD34), is strongly suggestive of the presence of diffuse glioma. The robustness of the results is underscored by the TMA model used for the study, as the limited amount of analysed tissue simulates well the settings of limited tissue sample. Furthermore, analysis of cell populations in consecutive sections from the same area sampled in TMA allows a more reliable assessment of protein coexpression than the whole section analysis. However, caution must be taken in the event of negative staining results: of 70 GBMS, one case was stained only with CD34, and two tumours stained only with SOX11. Furthermore, a significant subset of ependymomas and pilocytic astrocytomas was negative for all tested markers and thus the immunopanel is not helpful in excluding other subtypes of glioma that may rarely show infiltrative components in the tumour periphery. SOX11 is also almost constantly positive in mantle cell lymphoma and in occasional cases of diffuse large B-cell lymphoma [46], the most common subtype of primary CNS lymphoma. Thus, careful morphological and radiological correlation is needed for a proper diagnosis of limited brain biopsy samples, and the results must always be considered in the context of an appropriate antibody panel.

While the presented data show promising results for practical surgical neuropathology, there are several limitations of the study to be acknowledged. The most important limitation reflects the general limitations of immunohistochemistry. First, a proper calibration of antibody sensitivity is necessary to obtain the expected results [47], as illustrated by the experience with the EGFR antibody in previous studies. We provided a sensitivity threshold for EGFR aiming at high expressor cells. Furthermore, in our study CD34 was calibrated according to standardized NordiQC (www.nordiqc.org, accessed on 6 June 2022) suggestions. However, for setting up SOX11, INSM1, and MEOX2 assays, tissues with the strongest expression were chosen, given the lack of weak expressing tissues and general need to identify strong expressing cells of mantle cell lymphoma (SOX11), neuroendocrine tumours (INSM1), and endothelium (MEOX2). This must be considered for routine use and, ideally, immunoassays for this purpose should be validated with several samples of reactive and neoplastic glial tissue. Additionally, we acknowledge that despite a consensus of two independent observers, some weakly positive cases posed an interpretational challenge; these might be subject of diagnostic controversies in routine practice. Given the fact that most reactive tissues showed only weak positivity of tested markers, a conservative approach should be adopted in equivocal cases due to the possible clinical consequences (overtreatment of reactive lesion versus repeated biopsy). Finally, we are aware of the need of further validation of our results, preferably in another laboratory settings (i.e., with another immunohistochemistry platform and independently calibrated immunohistochemical assays) and an independent tumour cohort. It would be also beneficial to correlate the results with additional molecular methods, although a small content of tumour cells may effectively hamper this effort.

## Figures and Tables

**Table 1:** Antibodies and details of immunohistochemical reactions used in the study.

Antibody	Clone	Dilution	Manufacturer	Pretreatment	Incubation Time	Visualisation	Positive Control
GFAP	EP672Y	RTU	Ventana, Basel, Switzerland	Ventana CC1, 36 min.	16 min	Ventana ultraView	Brain
MEOX2	6A5	1:1000	Sigma Aldrich s.r.o., Prague, CR	Ventana CC1, 56 min.	32 min	Ventana OptiView	Appendix—HEV *
EGFR	31G7	1:100	Novus Biologicals, Ontario, Canada	Protease 3, 32 min	32 min	Ventana OptiView	Placenta
SOX11	MRQ-58	1:50	Cell Marque, Rocklin, CA, USA	Ventana CC1, 56 min.	32 min	Ventana OptiView	Foetal brain—subventricular zone
INSM1	A-8	1:100	SCBT Inc., Dallas, TX, USA	Dako PT Link High pH, 20 min.	30 min	DAKO EnVision FLEX	Pancreas
CD34	QBEnd10	1:50	Cell Marque, Rocklin, CA, USA	Ventana CC1, 20 min.	28 min	Ventana ultraView	Appendix

* HEV—high endothelial venules.

**Table 2:** Immunoreactivity of TMA cases for the individual immunohistochemical markers.

	Gliosis	Gliomas						*p*
		GBM	IDH1mt AA	OO	PA	EP	GG	
INSM1	2.3% (1/44)	81.6% (62/76)	35.3% (6/17)	82.4% (14/17)	12.5% (1/8)	0% (0/10)	80% (4/5)	<0.001 (χ^2^)
EGFR	0% (0/44)	50% (39/78)	23.5% (4/17)	17.6% (3/17)	0% (0/8)	0% (0/10)	0% (0/5)	<0.001 (χ^2^)
MEOX2	2.3% (1/43)	81% (64/79)	5.9% (1/17)	5.9% (1/17)	0% (0/8)	0% (0/10)	20% (1/5)	<0.001 (χ^2^)
SOX11	20.4% (9/44)	75% (57/76)	100% (17/17)	100% (17/17)	12.5% (1/8)	10% (1/10)	0% (0/4)	<0.001 (χ^2^)
CD34	11.6% (5/43)	25% (20/80)	0% (0/17)	0% (0/17)	12.5% (1/8)	20% (2/10)	60% (3/5)	0.39 (χ^2^)

Abbreviations: GBM—glioblastomas; AA—astrocytomas; OO—oligodendrogliomas; PA—pilocytic astrocytomas; EP—ependymomas; GG—gangliogliomas.

**Table 3:** mHS of studied markers in different tumour subsets of the TMA cohort.

	Gliomas			
	GBM	*IDH1mt* AA	OO	GG
INSM1	9 (2–23)	0 (0–0)	13.5 (±18.2)	3.9 (±3.5)
EGFR	0 (0–93.8)	0 (0–0)	0 (0–0)	0 (0–0)
MEOX2	7.25 (1–70.6)	0 (0–0)	0 (0–0)	0 (0–0)
SOX11	2 (0–17.9)	22.5 (2.5–45)	34.3 (±29.9)	0 (0–0)
CD34	0 (0–1)	0 (0-0)	0 (0-0)	7.4 (±10.5)

The data are presented as an average (±S.D.) for parametric data distribution or as a median (interquartile range, IQR) for the nonparametric data distribution (*p* < 0.05, Shapiro-Wilks test); Abbreviations: GBM—glioblastomas; AA—astrocytomas; OO—oligodendrogliomas; GG—gangliogliomas.

## Data Availability

The dataset used for the analysis is available in the form of a Supplementary File. Results of immunohistochemistry of individual tumours together with clinical data and the histology images (in the form of “*.svs” formatted image files) used and analysed during the current study are available from the corresponding author on reasonable request.

## Supplementary Materials

The following supporting information can be downloaded at: https://www.mdpi.com/article/10.3390/diagnostics13152546/s1, Table S1: Immunohistochemical results of the study cohort.
